# Dosimetric Impact of Source Displacement in GammaTile Surgically Targeted Radiation Therapy for Gliomas

**DOI:** 10.7759/cureus.38463

**Published:** 2023-05-02

**Authors:** Sook Kien Ng, Yong Yue, Kevin Shiue, Mitesh V Shah, Yi Le

**Affiliations:** 1 Radiation Oncology, Indiana University School of Medicine, Indianapolis, USA; 2 Neurological Surgery, Indiana University Health, Indianapolis, USA; 3 Radiation Oncology, Oklahoma Proton Center, Oklahoma City, USA

**Keywords:** gliomas, dosimetric changes, cavity shrinkage, seed displacement, gammatile

## Abstract

Background

This study aims to evaluate dosimetric changes that happened during the first month after GammaTile surgically targeted radiation therapy (STaRT) for gliomas due to Cesium-131 (Cs-131) seed displacement caused by cavity shrinkage in brain brachytherapy.

Methodology

In this study, 10 glioma patients had 4-11 GammaTiles placed along the resection bed after maximal safe resection during craniotomy. Each GammaTile is composed of four Cs-131 seeds embedded in a biodegradable collagen sponge to minimize seed movement and maintain seed-to-cavity surface distance. The Cs-131 seed positions were identified using VariSeed on day one. On day 30, post-implant computed tomography (CT) images and dosimetry parameters were calculated. An iterative closest point (ICP) algorithm was used to compute rigid transformation between the day one and day 30 seed clouds. The seed displacement was calculated after registration. The volume receiving 100% of the prescription dose (V100), the dose received by 90% of the planning target volume (D90_PTV), the planning target volume receiving 100% of the prescription dose (V100_PTV), and the dose to organs at risk (OARs) were calculated for both CT images to determine the dosimetric changes from any seed displacement.

Results

The mean seed displacement of 1.8 ± 1.0 mm for all patients was observed between day one and day 30. The maximum seed displacement for each patient ranged from 2.3 mm to 7.3 mm. The mean V100 difference between day one and day 30 was 2.5 cc (range = 0.5-6.5 cc). The mean D90_PTVs were 95.5% (range = 69.0%-131.0%) and 98.1% (range = 19.9%-149.0%) on day one and day 30, respectively. The mean V100_PTVs were 88.4% (range = 81.3%-99.1%) and 87.9% (range = 47.0%-99.7%) on day one and day 30, respectively. On day one, the brainstem dose was 63.5 Gy for one case and 28.1 Gy for another case; while on day 30, the brainstem dose was 55.8 Gy and 20.6 Gy for the same patients, contributing to 7.7 Gy (12.8%) and 7.5 Gy (12.5%) dose reductions to brainstem for these patients, respectively. Only two patients received a dose to the optic nerves (34.1 Gy and 5.2 Gy). There were small changes (1.8 Gy and 0.5 Gy, respectively) in the dose to optic nerves when comparing the dose calculated on day one and the dose calculated on day 30 CT images. The same two patients received 30.4 Gy and 6.8 Gy to the chiasm, respectively. Small changes in the dose to the chiasm (≤1.1 Gy) were noted between day one and day 30.

Conclusions

A maximum seed displacement of up to 7.3 mm and a mean seed displacement of 1.8 mm caused by cavity shrinkage were observed during the first month after GammaTile STaRT for gliomas. There were noticeable changes in dosimetry parameters. Changes in the doses to OARs, particularly the brainstem, were large (up to 12.8% of the prescription dose). These changes in dosimetry should be considered when evaluating treatment outcomes and planning future GammaTile treatments.

## Introduction

Radiation therapy after brain tumor surgery for high-grade gliomas has been shown to improve local tumor control [1,2]. However, delivering radiation therapy after surgery typically requires a waiting period of three to five weeks to allow for recovery. During this time, patients may experience tumor regrowth [3,4]. Brachytherapy has been explored in the past for the treatment of brain tumors with promising local control results [5-8]. While the success of multiple trials of brachytherapy with radioisotope seeds has been described, one factor that may limit the usage of brachytherapy in the management of brain metastases is the rate of radiation necrosis, which is associated with inconsistent radiation dose to the tumor bed [9,10]. Because the seeds were historically placed directly onto the brain surface, the area of the brain in direct contact with the seeds may receive supratherapeutic doses while the remaining brain may receive subtherapeutic doses. Previous studies have shown that to deliver a more homogeneous dose to the target and decrease the incidence of brain radionecrosis, a fixed separation between seeds should be used during implantation [11].

To address these challenges, the GammaTile (GT Medical Technologies, Tempe, AZ, USA) was designed as a permanently implantable collagen tile embedded with Cesium-131 (Cs-131) titanium-encased seeds that can be placed along the resection bed of malignant tumors [12,13]. Each GammaTile is composed of four Cs-131 seeds embedded in a biodegradable collagen material at fixed positions to minimize seed movement and maintain a seed-to-cavity surface distance. Embedding Cs-131 seeds in biodegradable collagen tiles that adhere to the walls of resection beds minimizes seed migration, provides fixed separation between seeds, and allows for the delivery of more homogeneous doses [11,14]. However, any cavity shrinkage that occurs after the resection may cause the collagen tiles to shift or change shape, potentially leading to seed displacement [13], even though the collagen tiles remain adhered to the cavity wall.

This study aimed to evaluate any dosimetric changes that occurred during the first month following GammaTile surgically targeted radiation therapy (STaRT) for gliomas due to Cs-131 seed displacement caused by cavity shrinkage.

This study was previously presented as a meeting abstract and an e-poster at the 2022 American Association of Physicists in Medicine Annual Meeting held in Washington, DC.

## Materials and methods

A total of 10 glioma patients who underwent craniotomy for glioma resection at our institution between 2021 and 2022 and were subsequently treated with GammaTile STaRT at the time of surgery were identified for this study. The treatment was administered as part of an institutional review board-approved trial (approval number: 11857). Four of the patients were males and six were females, with ages ranging from 33 to 68 years and a mean age of 51 years. There were two grade II gliomas, four grade III gliomas, and four grade IV gliomas among these cases. Patients had 4-11 GammaTiles placed along the resection bed after maximal safe resection during craniotomy. One day after tumor resection and the placement of GammaTiles, all patients received post-implant imaging (both computed tomography (CT) and magnetic resonance imaging (MRI)) to assess the extent of resection and any postoperative hematomas or new mass lesions. Subsequently, all patients received post-implant imaging 30 days after the placement of GammaTiles on their one-month follow-up visits. Post-implant CT images were imported into the brachytherapy planning software, VariSeed 9.0 (Varian Medical System Inc. Palo Alto, CA 94304). The radioisotope was configured in brachytherapy planning software by entering seed information, such as half-life, source geometry (line source), calculation model (line source), dose rate constant, kerma-to-activity conversion factor, radial dose function, and anisotropy function for modeling the two-dimensional line source of the IsoRay Medical model Cs-1 Rev2 Cs-131 seed [15]. The Cs-131 seeds were identified on day one and day 30 CT images by experienced medical physicists using the seed finder feature in the VariSeed 9.0 planning software using manual adjustment if necessary. The collection of all seed positions of each implant in three dimensions was exported as a seed cloud.

The day one and day 30 seed clouds were spatially aligned using an iterative closest point (ICP) algorithm. The rigid transformation was used to register the two seed clouds. To perform the task of fitting data points to model points, the ICP algorithm uses the command [R, T] = icp(model, data). The inputs for the ICP algorithm include the following two matrices: (1) The model matrix, which contains the model points [Pm_1, Pm_2, ..., Pm_nmod]; and (2) The data matrix, which contains the data points [Pd_1, Pd_2, ..., Pd_ndat]. Both matrices should be properly formatted per the requirements of the ICP algorithm. The ICP algorithm produces a rotation matrix R and a translation vector T as output, which describe the transformation needed to align the data points with the model points. Therefore, new data = R*data + T, where new data are transformed data points to fit the model.

**Figure 1:**
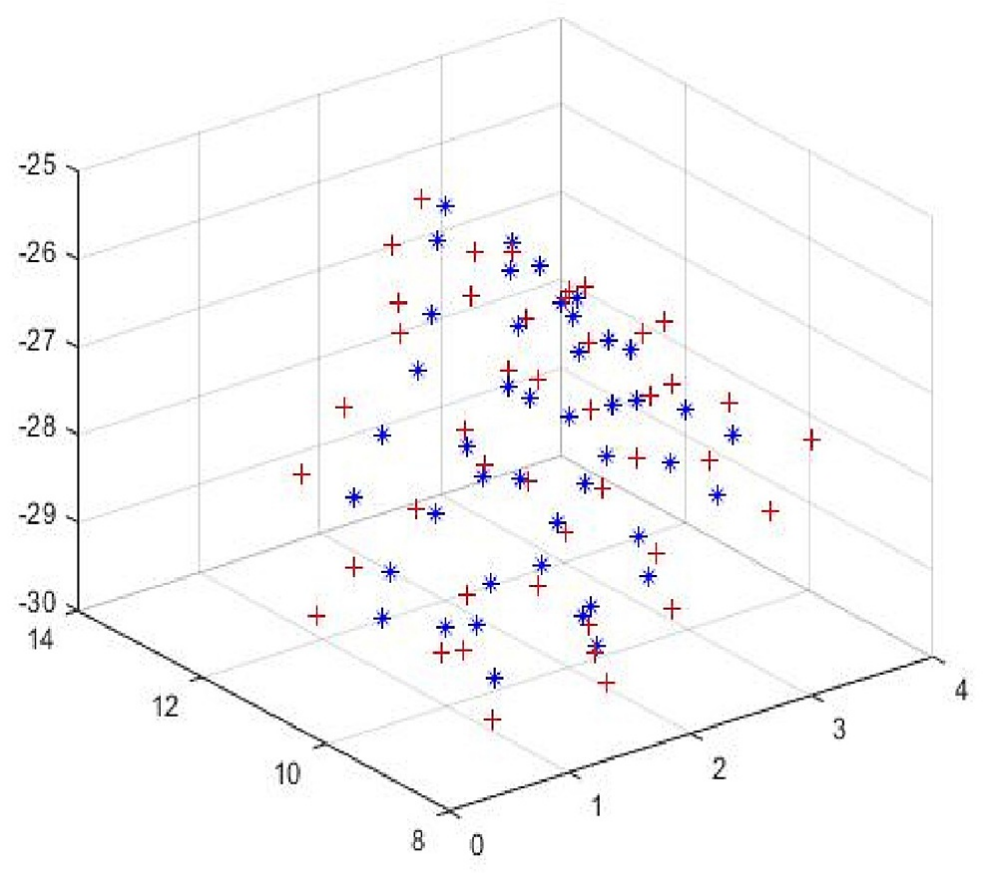
Registration of day one and day 30 seed clouds. Day one and day 30 seed clouds are spatially aligned using the ICP algorithm. The rigid transformation was used to register the two seed clouds. The day one seed cloud is shown in red and the day 30 seed cloud is shown in blue for one of the cases. ICP = iterative closest point

Specifically, we used [TR, TT] = icp(d30CTseeds, d1CTseeds) to align the day one CT seed cloud with the day 30 seed cloud. Next, we transformed the coordinates of day one seed locations to the day 30 coordinate system. Once the seed locations from both seed clouds were marched in pairs, we calculated the displacement between day 30 and day one. Finally, we used the above methods to calculate the average and maximum seed displacement. Figure 1 shows the two registered seed clouds of one of the cases analyzed. The mean seed displacement (the average value of seed displacement for the entire seed collection in each implant) and the maximum seed displacement between day one and day 30 for each patient’s dataset were calculated after registration.

Information about the two-dimensional line source of the IsoRay Medical model Cs-1 Rev2 Cs-131 seed was entered into the brachytherapy planning software to configure the radioisotope. The parameters entered included the half-life, source geometry (line source), calculation model (line source), dose rate constant, kerma-to-activity conversion factor, radial dose function, and anisotropy function, all of which were described in TG-43 Supplement 2 [15]. The radiation doses were calculated per the American Association of Physicists in Medicine TG-43 protocol using the seed position identified in the day one CT images. In addition, using the updated seed positions obtained on the day 30 CT images without accounting for decay, the dose distributions for each implant were recalculated on the day 30 post-implant CT images. The resection cavity was outlined using seeds as a surrogate. The residual tumor, identified as contrast-enhanced areas in the post-implant T1-weighted MRI with contrast images, was contoured by radiation oncologists and defined as the gross target volume of residual tumor (GTVr). The planning target volume (PTV) is defined as the sum of GTVr and a 5 mm expansion of the cavity volume. The goal of GammaTile STaRT is to deliver a radiation dose of 60 Gy to the PTV. The volume receiving 100% of the prescription dose (V100), the percent of the prescription dose received by 90% of the PTV (D90_PTV), and the volume of the PTV receiving 100% of the prescription dose (V100_PTV) were calculated and analyzed for both day one and day 30 datasets to determine any dosimetric changes due to seed displacement. The radiation doses to organs at risk (OARs), such as the brainstem, optical nerves, and chiasm, were also calculated.

## Results

Table 1 shows seed displacement and dosimetry parameters for each patient. The mean seed displacement of 1.8 ± 1.0 mm for all patients was observed between day one and day 30. The maximum seed displacement for each patient ranges from 2.3 mm to 7.3 mm. The maximum seed displacement was large in a few cases, more than 4 mm, in five out of 10 cases (50% of all patients in this study). Three cases experienced a seed displacement that resulted in a volume change of more than 3.0 cc in V100 between day one and day 30. The mean V100 difference between day one and day 30 was 2.5 cc (range = 0.5-6.5 cc). A D90_PTV change between day one and day 30 of more than 20% was observed in five cases. The mean D90_PTVs were 95.5% (range = 69.0%-131.0%) and 98.1% (range = 19.9%-149.0%) on day one and day 30, respectively. While the mean V100_PTV changes were small (mean value = -0.5%), a PTV coverage reduction of 36% was observed on the day 30 post-implant CT in one case. The mean V100_PTVs were 88.4% (range = 81.3%-99.1%) and 87.9% (range = 47.0%-99.7%) on day one and day 30, respectively.

**Table 1:** Seed displacement and dosimetric changes that happened during the first month after GammaTile STaRT. Mean and maximum seed displacements for each patient were calculated after registration of day one and day 30 seed clouds. In addition, PTV volume, V100, and PTV coverage (D90_PTV, V100_PTV) were calculated using seed positions identified in day one and day 30 CT images to determine dosimetric changes due to seed displacement. STaRT = surgically targeted radiation therapy; PTV = planning target volume; V100 = volume receiving 100% of the prescription dose; D90_PTV = dose received by 90% of the planning target volume; V100_PTV = planning target volume receiving 100% of the prescription dose; CT = computed tomography

Pt	Mean seed displacement (mm)	Maximal seed displacement (mm)	PTV_vol_D1 (cc)	PTV_vol_D30 (cc)	PTV_vol_diff (cc)	D90_PTV_D1 (%)	D90_PTV_D30 (%)	D90_PTV_diff (%)	V100_PTV_D1 (%)	V100_PTV_D30 (%)	V100_PTV_diff (%)	V100 _D1 (cc)	V100 _D30 (cc)	V100_diff (cc)
1	1.9	2.7	35.6	30.1	5.5	99.5	103.0	3.5	90.0	91.7	1.7	35.7	33.7	2.0
2	1.2	3.4	53.6	69.8	16.2	88.0	19.9	-68.1	83.0	47.0	-36.0	48.7	46.8	2.0
3	2.7	4.4	55.9	44.9	11.0	96.3	118.5	22.2	87.9	97.6	9.7	56.6	53.5	3.1
4	2.5	6.2	78.8	50.2	28.6	70.0	36.0	-34.0	81.3	81.4	0.1	77.2	73.6	3.7
5	1.7	4.1	60.0	53.4	6.6	102.3	107.0	4.7	90.7	92.4	1.7	70.0	67.3	2.6
6	1.1	3.2	29.1	23.5	5.6	131.0	143.0	12.0	99.1	99.7	0.6	42.6	41.3	1.3
7	0.7	3.3	25.9	27.5	1.6	96.0	89.0	-7.0	87.9	86.3	-1.6	26.7	26.2	0.5
8	3.6	7.3	84.5	58.2	26.3	121.0	149.0	28.0	96.7	99.5	2.8	105.0	98.5	6.5
9	0.6	2.5	28.6	24.3	4.3	82.0	100.0	18.0	85.4	90.0	4.6	33.7	32.8	0.9
10	2.3	4.5	87.3	61.9	25.4	69.0	116.0	47.0	81.7	93.5	11.8	92.5	89.8	2.8
Mean	1.8	4.2	53.9	43.4	13.1	95.5	98.1	2.6	88.4	87.9	-0.5	58.9	56.3	2.5

The doses to the brainstem, optic nerves, and chiasm were calculated and are shown in Table 2. The dose calculation performed on day one post-implant CT images showed that six patients received more than 15 Gy to the brainstem. Only two patients received a dose to the optic nerves and the chiasm. One patient received 34.1 Gy to the optic nerves and 30.4 Gy to the chiasm. The other patient received 5.2 Gy to the optic nerves and 6.8 Gy to the chiasm. Four out of the 10 patients in this study did not receive a dose to OARs. Comparing the OAR dose on day one and day 30, we observed a reduction in dose to OARs on day 30 for most cases. This is due to cavity shrinkage that moved the seeds away from the OARs and thus reduce the dose to OARs. A larger dose reduction was observed in the brainstem dose. The radiation dose calculated on the day one post-implant CT images showed the brainstem dose was 63.5 Gy for one case and 28.1 Gy for another case. The radiation dose calculated on the day 30 CT images showed brainstem dose was 55.8 Gy and 20.6 Gy for the same patients, contributing to a 7.7 Gy (12.8%) and a 7.5 Gy (12.5%) dose reduction to the brainstem for these patients, respectively. While there were small changes (1.8 Gy in one case and 0.5 Gy in the other) in the dose to the optic nerves when comparing the dose calculated in the day one and day 30 CT images, increments of the dose to optic nerves were noted in the day 30 CT images. Only marginal changes (≤1.1 Gy) of the dose to chiasm were noted between day one and day 30.

**Table 2:** Dose to organs at risk calculated using day one and day 30 post-implant CT images and the differences between the two datasets. Dose to the brainstem, optic nerves, and chiasm were calculated for day one and day 30 post-implant CT images. A large dose reduction on day 30 was observed in the brainstem dose for a few patients. There was only a small change in dose to optic nerves and chiasm between day one and day 30. Four out of the 10 patients in this study did not receive any dose to the OARs. CT = computed tomography; OARs = organs at risk

Pt	Brainstem_D1 (Gy)	Brainstem_D30 (Gy)	Brainstem_diff (Gy)	Optic _nerves_D1 (Gy)	Optic_nerves_D30 (Gy)	Optic_nerves_diff (Gy)	Chiasm_D1 (Gy)	Chiasm_D30 (Gy)	Chiasm_diff (Gy)
1	0.0	0.0	0.0	0.0	0.0	0.0	0.0	0.0	0.0
2	0.0	0.0	0.0	0.0	0.0	0.0	0.0	0.0	0.0
3	20.6	14.3	-6.3	0.0	0.0	0.0	0.0	0.0	0.0
4	63.5	55.8	-7.7	0.0	0.0	0.0	0.0	0.0	0.0
5	15.5	12.1	-3.4	0.0	0.0	0.0	0.0	0.0	0.0
6	0.0	0.0	0.0	0.0	0.0	0.0	0.0	0.0	0.0
7	28.1	20.6	-7.5	34.1	35.9	1.8	30.4	30.2	-0.2
8	52.0	45.3	-6.7	0.0	0.0	0.0	0.0	0.0	0.0
9	0.0	0.0	0.0	0.0	0.0	0.0	0.0	0.0	0.0
10	23.1	26.4	3.3	5.2	5.7	0.5	6.8	7.9	1.1

## Discussion

**Figure 2:**
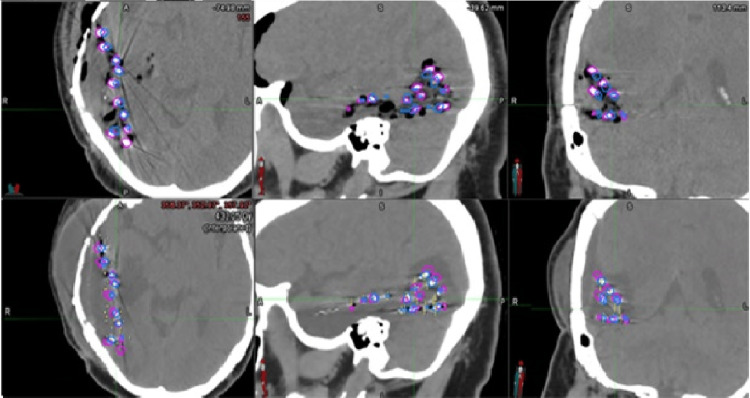
Seed positions identified on day one and day 30 post-implant CT images superimposed on both CT images. Significant maximum seed displacement was observed in a few cases. Seed positions identified on day one and day 30 post-implant CT images superimposed on day one and day 30 post-implant CT images for a patient are shown here as an example. Day one CT is shown in the top row while day 30 CT is shown in the bottom row with seeds of day one shown in pink and day 30 in blue, overlaying in both image sets.

It is worth noting that the PTV coverage (D90_PTV and V100_PTV) was caused by the combined effect of changes in PTV volume/shape due to resection cavity shrinkage and the effect of seed displacement. Contrary o a previous study done by Pinnaduwage et al. [16] that reported a small (≤4.3 mm) maximum seed displacement and small dose difference observed in their study, a larger maximum seed displacement was observed in a few cases in our study. The seed positions of day one and day 30 superimposed on the day one and day 30 CT images for a patient are shown in Figure 2 as an example. While the maximum seed displacement was large in a few cases, we were not able to quantify the PTV coverage changes due to either the seed displacement or cavity shrinkage separately. Because the post-implant images were only acquired at two time points, we are unable to ascertain when these changes in cavity dimension and seed displacements occurred, nor the rate of the changes (gradually over the period of a month or suddenly during the first few days after surgery/implantation). Further studies with larger numbers of patients are necessary to determine the optimal timing for post-implant MRI and CT scans for implanted GammaTile patients.

One limitation of this study is that the post-implant images were acquired only at two time points (on day one and day 30 post-implant). This study evaluated dosimetric changes that occurred during the first month after GammaTile STaRT for gliomas due to seed displacement caused by cavity shrinkage with the assumption that the cavity shrinkage and seed displacement observed mostly happened during the first few days after tumor resection and GammaTile placement when the dose delivery of Cs-131 seeds was high (88.3% of the dose from Cs-131 is delivered within the first month post-implant). This assumption is based upon previous findings that reported cavity shrinkage occurring immediately after surgery, and relatively minimal cavity shrinkage was observed after the first few days of tumor resection [13,17]. While several studies indicate that shrinkage occurs consistently over time in a significant proportion of patients [11,18,19], Atalar et al. [13] reported significant cavity shrinkage occurring immediately after surgery (postoperative days 0-3) in a surgery and post-resection cavity stereotactic radiosurgery population, without further significant change over the remainder of the 33-day follow-up period. Jarvis et al. [17] also suggested that the most significant cavity size changes occurred in the earliest postoperative period. Alghamdi et al. [20] found an average cavity volume reduction of 22.5% at a median duration of four weeks after surgery, with most changes occurring within three weeks. After surgery, the surgical bed is dynamic and prone to significant changes in the dimensions of the resection cavity. The resection cavity volume may undergo several changes as the resection space contracts, surrounding edema fluctuates, and the residual tumor volume changes. It is difficult to monitor these changes with a minimal amount of reasonable re-imaging. The assumption that the cavity shrinkage observed mostly occurred during the first few days after tumor resection serves as the worst-case scenario of dosimetric changes that could occur in these cases.

## Conclusions

In this study, up to 7.3 mm of seed displacement caused by cavity shrinkage was observed during the first month after GammaTile STaRT for gliomas. Cavity shrinkage also caused a large change in PTV coverage due to changes in the resection cavity volume and shape that caused the seed displacement. The volume change on V100 was noticeable, and the PTV coverage changes were large in a few cases. Changes to the OARs dose, particularly the brainstem, were substantial. These changes in dosimetry should be considered when evaluating treatment outcomes and planning future GammaTile treatments. However, the number of patients included in this study was small, and the post-implant images were only acquired at two time points. To better evaluate the changes in radiation dosage resulting from Cs-131 seed displacement due to cavity shrinkage in brain brachytherapy, it is necessary to conduct additional studies. These studies should involve a larger patient group and utilize post-implant images taken at multiple time points. This is important because changes in the resection cavity can cause the implant to shift, which can impact the accuracy of the radiation dose.
